# Selenium and Mammalian Uterine Health: A Comprehensive Review

**DOI:** 10.3390/biology14091295

**Published:** 2025-09-19

**Authors:** Ruoning Wu, Xiaohan Li, Zhaoming Li, Jilong Luo, Ziwei Zhang, Mengyao Guo

**Affiliations:** College of Veterinary Medicine, Northeast Agricultural University, Harbin 150030, China; s240601078@neau.edu.cn (R.W.); 19544557995@163.com (X.L.); lizhaoming1210@126.com (Z.L.); luojilong@neau.edu.cn (J.L.); zhangziwei@neau.edu.cn (Z.Z.)

**Keywords:** selenium, uterine, animal production, animal health, oxidative stress, essential nutrients

## Abstract

Selenium (Se) is an essential trace element for animals and plays an important role in maintaining various physiological functions. In recent years, more and more studies have focused on the relationship between selenium and mammalian uterus health. This review comprehensively summarizes the current research progress in this field and introduces the physiological functions of selenium, including its antioxidant, anti-inflammatory, and immune regulatory functions. It also analyzed the role of selenium in uterine development, maintaining pregnancy, preventing and treating uterine diseases from multiple aspects. In addition, factors affecting animal selenium utilization and appropriate selenium supplementation strategies were also introduced. Understanding the relationship between selenium and animal uterine health is of great significance for improving animal reproductive performance, ensuring offspring health, and promoting sustainable development of the animal husbandry.

## Simple Summary

Selenium is a trace element widely found in living organisms. Selenium deficiency can cause abnormal growth in animals. In this review, we understand how selenium can prevent uterine disease, ensure uterine development, and maintain pregnancy, thereby providing new ideas for the animal breeding industry. We summarize the different ways selenium acts on the uterus, hoping to provide ideas and methods for selenium supplementation in production.

## 1. Introduction

Selenium is an essential trace element with the symbol Se, atomic number 34 and relative atomic mass 78.96. It has been proven to have a wide range of physiological functions in animals, including antioxidant, anti-inflammatory, anti-apoptotic, and immunomodulatory effects [1,2]. Research has shown that selenium can exert antioxidant effects by activating selenium containing enzyme systems such as glutathione peroxidase (GPx), effectively clearing excess reactive oxygen species (ROS) in the body and reducing oxidative stress damage [3]. Selenium supplementation can better relieve hyperthyroidism and improve mild thyroid eye disease [4]. Moreover, selenium can regulate the body’s inflammatory response, inhibit the overexpression of pro-inflammatory factors, and exert anti-inflammatory effects [5]. Dietary supplementation with selenium reduces the accumulation of toxic (Cd and As) and potentially toxic (Cr and Ni) trace elements in rabbits. However, excessive intake may negatively affect essential trace elements [6]. In addition, selenium also has a regulatory effect on the immune system, which can enhance the activity of immune cells and maintain the immune balance of the body [1].

The uterus is an important reproductive organ in female mammalians, responsible for functions such as embryo implantation, maintaining pregnancy, and fetal development, and plays an indispensable role in the reproductive process. Therefore, maintaining such a good physiological condition of the uterus is essential for ensuring the smooth and secure reproductive process of female animals. Thus, the essential tasks of population continuity and reproduction are realized.

In recent years, with the continuous deepening of research, the relationship between selenium and animal uterine health has gradually become a research hotspot. More and more evidence shows that selenium is closely related to animal uterine health. Adequate selenium levels can promote uterine development, improve uterine function during pregnancy, and help prevent and treat uterine diseases [7].

In animal husbandry, the bioavailability of selenium is regulated by multiple factors, among which the source and type of selenium and the interaction mechanism with nutrients are key influencing factors [8]. The metabolic pathway and utilization efficiency of selenium in animal bodies depend on its chemical form. Inorganic selenium sources (such as sodium selenite) have been widely used in animal feed due to their low cost, but this type of selenium source requires a complex transformation process in the animal body to be absorbed and utilized [9,10], and excessive intake can easily lead to the risk of selenium poisoning [11]; In contrast, organic selenium sources have the advantages of being safer and more efficient [12] (Table 1). Selenomethionine, as a typical organic selenium source, can be directly integrated into the protein primary structure through the methionine transport mechanism [13], resulting in a utilization rate of up to 85–95% in animal bodies [10,14]; selenium yeast [15] is rich in various organic selenium compounds such as selenomethionine [16] and selenocysteine [17], which significantly enhance the absorption efficiency of selenium through the synergistic effect of gut microbiota [18,19]. Studies have shown that the addition of organic selenium can elevate selenium levels in the intestinal flora and alleviate Alzheimer’s disease [20]; embedding probiotics into selenium nanolocations synergistically eliminates ROS, regulates gut microbiota, and alleviates ulcerative colitis [21]. However, there are significant differences in the metabolic kinetics of different organic selenium sources in animals, which leads to diverse biological activities and functions [22]. Several studies have proven that feeding selenium-enriched feeds can enhance the texture of pork and improve meat color and brightness [23,24]. The addition of selenium-enriched yeast increases the pH of meat after slaughter and slows down the rate of meat spoilage [25]. Therefore, we would like to see more research exploring whether feeding selenium-enriched diets can lead to improved performance of female animals. This review aims to comprehensively summarize the research progress on the relationship between selenium and animal uterine health, providing a theoretical basis for improving animal reproductive performance and promoting the development of the animal husbandry industry. Furthermore, it provides future perspectives, offering direction for future research to fill gaps in practical therapeutic approaches for uterine diseases.

## 2. Physiological Functions of Selenium in Animals

### 2.1. Antioxidant Function

**Figure 1:**
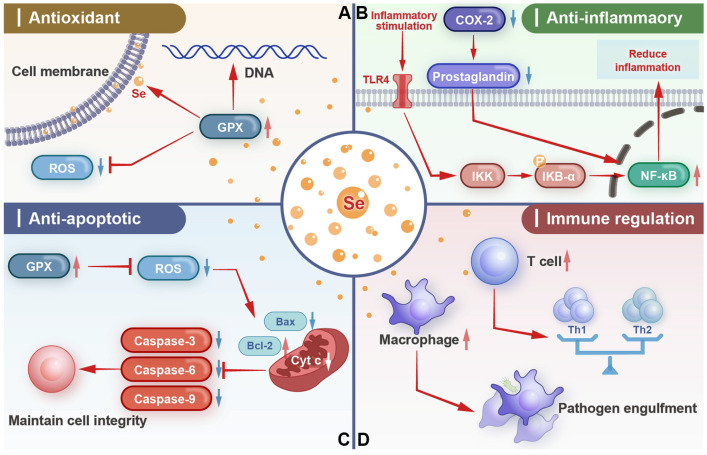
Physiological roles played by selenium in the animal body. (**A**) Antioxidant effects of selenium; (**B**) Anti-inflammatory effects of selenium; (**C**) Anti-apoptotic effects of selenium; (**D**) Immunomodulatory effects of selenium.

Selenium is an important component of several antioxidant enzymes, such as GPx. GPx catalyzes the reduction of hydrogen peroxide and lipid hydroperoxides to water and corresponding alcohols [30], thereby scavenging reactive oxygen species (ROS) and protecting cells from oxidative damage [31]. In the uterine environment, oxidative stress can be induced by various factors such as inflammation, pregnancy-related physiological changes, and environmental toxins [32,33,34]. Selenium-containing GPx can effectively reduce the levels of ROS and nitric oxide (NO) in uterine cells, reduce the production of free radicals [35,36,37], and protect the integrity of cell membranes, DNA, and proteins, and maintain normal uterine function [32]. For example, in cows or mice with endometritis, the increase in ROS levels can damage endometrial cells [38,39]. Supplementing dairy cows with sodium selenite (5 mg/day) and vitamin E (2 g/day) for 10 days can increase the concentration of glutathione peroxidase in uterine tissue [40]. Supplementing mice with 0.15 mg/kg selenium can enhance the activity of GPx, reduce oxidative stress, and promote the repair of damaged endometrial cells [41,42] (Figure 1A).

### 2.2. Anti-Inflammatory Function

Selenium also plays a role in regulating the inflammatory response in animals. It can modulate the expression and activity of various inflammatory mediators (Figure 1B). One of the key mechanisms involves the inhibition of the nuclear factor-kappa B (NF-κB) signaling pathway [43,44]. When the uterus is exposed to pathogens or other stressors, NF-κB is activated and transferred to the nucleus [45,46], where it initiates transcription of genes encoding pro-inflammatory cytokines such as tumor necrosis factor-α (TNF-α), interleukin-1β (IL-1β), and interleukin-6 (IL-6) [47]. Selenium can interfere with the activation process of NF-κB. It can enhance the inhibitory effect of IκB (NF-κB inhibitor) on NF-κB [48], preventing its activation and nuclear translocation [49]. As a result, the production of pro-inflammatory cytokines is reduced, alleviating inflammation of the uterus (Figure 1B). In addition, selenium can regulate the expression of cyclooxygenase-2 (COX-2) [50], a key enzyme in prostaglandin synthesis [51,52]. By reducing the expression of COX-2, selenium can further alleviate the inflammatory response in uterine tissue [53].

### 2.3. Anti-Apoptotic Function

Cell apoptosis is a programmed cell death, which plays an important role in maintaining tissue homeostasis [54]. Selenium can regulate apoptosis in uterine cells. In normal physiological conditions, selenium may help maintain a balance between cell proliferation and apoptosis in the uterus [55,56]. However, selenium can protect uterine cells from excessive apoptosis by upregulating the expression of anti-apoptotic genes during oxidative stress or inflammation, such as B-cell lymphoma 2 (Bcl-2), or downregulating the expression of pro-apoptotic proteins such as Bcl-2-associated X protein (Bax) [57,58]. By changing the ratio of Bcl-2/Bax, selenium can inhibit the activation of caspases [59], which are key enzymes in the apoptotic pathway. For example, in the bovine endometrial epithelial cells (BEECs), with lipopolysaccharide (LPS) induced inflammation related to apoptosis. Adding selenium can increase the ratio of Bcl-2/Bax and reduce the activity of caspase-3, thereby inhibiting cell apoptosis [60]. Similarly, selenium can also alleviate zearalenone induced apoptosis of porcine endometrial epithelial cells [61]. Selenium can also interfere with the mitochondrial pathway of apoptosis. It can prevent the release of cytochrome C from the mitochondria into the cytosol [58,62], which is a crucial step in the activation of the caspase cascade [63]. GPX4 protects cells from mitochondrial pathway-mediated apoptosis by blocking the mitochondrial release of cytochrome C, inactivating caspase 3, and inhibiting hydrogen peroxide production [64] (Figure 1C). By blocking cytochrome C release, selenium can effectively prevent apoptosis of uterine cells under stress conditions [65,66].

### 2.4. Immune-Regulatory Function

Selenium is essential for the normal development and function of the immune system in animals, as it affects the proliferation, differentiation, and function of immune cells such as lymphocytes and macrophages [2,67,68]. Selenoprotein is involved in the regulation of immune responses, including antibody production, cell-mediated immunity, and innate immune responses. Macrophages are the first line of defense for the uterus against pathogens [69], and selenium can activate macrophages in uterine tissue [40,57,70,71]. Macrophages rich in selenium exhibit stronger ability to engulf and eliminate invading bacteria and other pathogens [72]. Moreover, T lymphocytes can differentiate into different subgroups, such as Th1 and Th2 cells, and selenium can regulate this differentiation process [73]. A high-selenium diet can alleviate the activation of F4/80 macrophages in the alveoli of mice and the activation of pulmonary CD4+ and CD8+ T cells, and can be used for the treatment of Keshan disease [74]. Selenium maintains the dynamic balance of Th1/Th2 immune responses to maintain uterine health and ensure that the immune system can alleviate uterine tissue damage caused by pathogen attacks [75,76,77] (Figure 1D).

## 3. Selenium and Uterine Development

### 3.1. Effects on Uterine Morphology and Structure

Selenium in mammals is primarily obtained from the diet. Both monogastric animals and humans can digest and absorb dietary selenium efficiently, with human utilization rates ranging from 56% to 91% [78]. However, ruminants exhibit significantly reduced utilization of dietary selenium [79], primarily because selenides (Se^2−^) are not absorbed in the rumen and can only be absorbed in the small intestine [80]. We summarized the selenium requirements of different mammals and the maximum doses used (Table 2).

During the growth and development of female animals, the level of selenium has an impact on the development of uterine morphology and structure [81]. Selenium is required for the normal development of the endometrium, myometrium and blood vessels. In the study of ruminants, selenium deficiency has been shown to lead to abnormal uterine development [82], such as thinning of the endometrial layer and decreased density of smooth muscle cells in the muscular layer [83]. In contrast, appropriate selenium supplementation can promote the proliferation and differentiation of uterine cells, thereby making the mice uterine structure more developed and functional [84,85]. For example, in growing cows, it has been found that adding selenoprotein to the diet can increase the thickness of the endometrium and improve the tissue structure of the uterine glands, which may help improve the reproductive performance of cows [86,87].

Selenium promotes the proliferation of uterine cells and has an impact on the development, maintenance of physiological functions, and repair of the uterus after injury [88]. The mechanism of selenium induced cell proliferation is complex [89]. Selenium can regulate the expression of genes related to cell cycle progression. For example, it can upregulate the expression of cyclin dependent kinases (CDKs) and cyclin [90,91], which are key regulatory factors of the cell cycle [92]. Selenium supplementation has been shown to increase the expression of cyclin D1 and CDK4 in cells [91,93], promoting the transition of cells from the G1 phase to the S phase of the cell cycle [94,95]. In addition, selenium can activate signaling pathways such as the PI3K/Akt/mTOR pathway whose activation can phosphorylate downstream effectors, leading to protein synthesis and cell growth [96]. Selenium supplementation has been found to activate the PI3K/Akt/mTOR pathway and promote cell proliferation in bovine endometrial cells [86,96,97]. Selenium can also enhance the expression of growth factors and their receptors, such as epidermal growth factor (EGF) and its receptor (EGFR) [98]. The EGF/EGFR signaling pathway is closely related to cell proliferation [99]. Selenium can increase the levels of EGF and EGRF, stimulate the EGF/EGFR-mediated signaling cascade, and further promote uterine cell proliferation [100]. By leveraging selenium’s ability to promote cell proliferation, it can more effectively repair damage.

### 3.2. Molecular Mechanisms and Signaling Pathways

**Figure 2:**
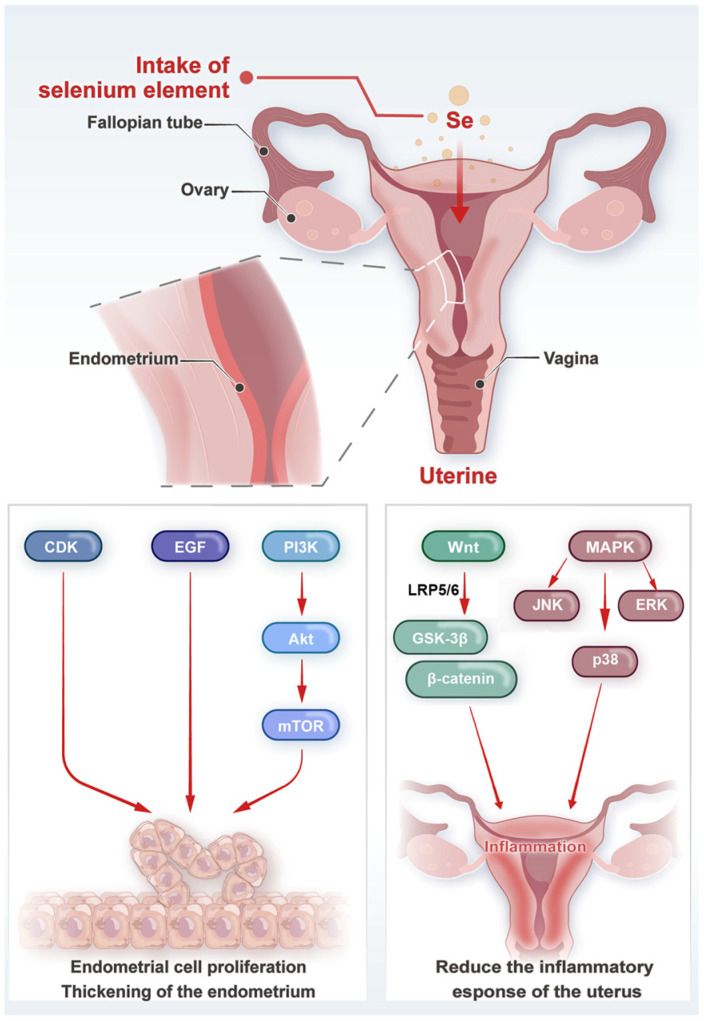
The effect of selenium on uterine development.

Selenium affects uterine development by regulating the expression of genes associated with cell proliferation, differentiation, and extracellular matrix remodeling. The key to this is the regulation of growth factor signaling. Insulin-like growth factor 1 (IGF-1) [101,102], a key growth factor for uterine development, is a cell proliferation regulator [103]. Selenium supplementation increases the expression of IGF-1 and its receptor (IGF-1R) in uterine cells [104], leading to binding of IGF-1 to IGF-1R and activation of downstream signaling pathways [105,106], such as the PI3K/Akt pathway [107] (Figure 2). Activation of PI3K and Akt regulates cell proliferation, metabolism, and survival by phosphorylating multiple downstream targets [108,109].

Also, selenium can affect uterine development through other cell signaling pathways. In addition to the PI3K/Akt/mTOR pathway mentioned above, these include the Wnt/β-catenin signaling pathway and the mitogen-activated protein kinase (MAPK) pathway [110,111,112,113] (Figure 2). The Wnt/β-catenin pathway plays a key role in cell proliferation, differentiation, and especially embryonic development [114]. Activation of this pathway stabilizes β-catenin levels and promotes its nuclear translocation [115,116]. Upon entering the nucleus, β-catenin binds to transcription factors and regulates the expression of target genes [117,118]. In uterine cells, selenium promotes cell proliferation and expression of genes related to uterine function through activation of the Wnt/β-catenin pathway. Studies have shown that selenium can activate the PI3K/AKT and Wnt/β-catenin pathways to promote the proliferation of bovine endometrial stromal cells (BESCs), which contributes to the repair of the endometrium after delivery [60,119]. Selenium also modulates the activity of different members of the MAPK family, such as extracellular signal-regulated kinase (ERK), c-Jun N-terminal kinase (JNK), and p38 MAPK [59,120]. Activation of these kinases regulates a variety of cellular processes, including cell proliferation, apoptosis, and inflammation [121,122]. Studies have shown that hydrogels with added selenium nanoparticles can reduce inflammation by inducing macrophage polarization toward the M2 type via the MAPK pathway [123]. Selenium induces activation of the ERK pathway [124], which promotes cell proliferation by phosphorylating transcription factors of genes involved in the regulation of the cell cycle [125]. Selenium inhibits p38 MAPK phosphorylation even under high cortisol levels, enhancing the anti-inflammatory capacity of primary bovine endometrial stromal cells [126]. On the other hand, selenium-activated p38 MAPK pathway reduces the expression of pro-inflammatory cytokines and alleviates inflammation in uterine cells [127,128,129]. In contrast, selenium deficiency leads to elevated ROS levels in human uterine smooth muscle cells and increased levels of p-P38 and p-JNK gene expression, ultimately leading to increased apoptosis and necrotic apoptosis [130].

Besides the above-mentioned cell signaling pathways, selenium also affects the expression of genes encoding extracellular matrix components such as collagen and fibronectin [131,132]. By regulating the synthesis and degradation of these extracellular matrix proteins, selenium sustains the structure and function of the uterus during development.

**Table 2:** Selenium requirements in different mammalian species and maximum doses used.

Species	Recommended Dietary Se Intakes [80]	Use the Maximum Dose
Rats	0.15 mg/kg Diet	selenite/selenate LOAELlethality ^2^: 1.2 mg Se/kg bw/day [133]
Mice	0.15 mg/kg Diet	selenocystine LOAELlethality ^2^: 15 mg Se/kg bw/day [133]
Pigs	0.15–0.3 mg/kg Diet	4 mg/kg
Dogs	0.35 mg/kg Diet	5 mg/kg
Cats	0.30 mg/kg Diet	5 mg/kg
Beef cattle	0.1 mg/kg DMI ^1^	3–8 mg/kg
Dairy cows	0.3 mg/kg DMI ^1^	5 mg/kg
Sheep & Goats	0.1 mg/kg DMI ^1^	Not available

^1^ DMI, dry matter intake; ^2^ LOAEL, assess dose levels at which toxicity occurs.

## 4. Selenium and Uterine Function During Pregnancy

### 4.1. Embryo Implantation

**Figure 3:**
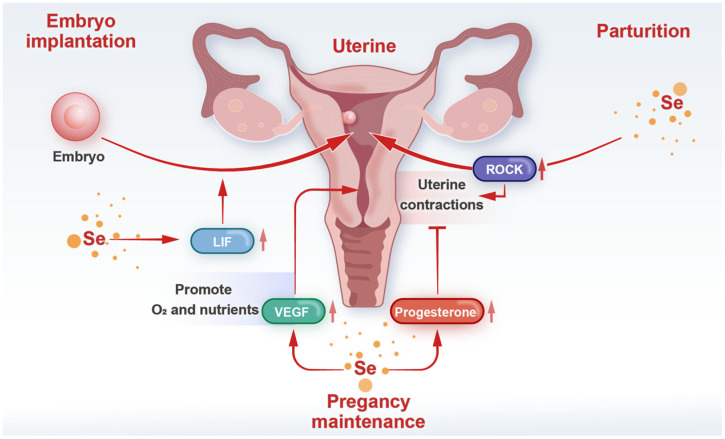
The role played by selenium in the uterus of pregnant animals.

Implantation is a critical step in the pregnancy process and an important stage in early embryonic development [134], during which the uterus must create a suitable microenvironment for embryo attachment and invasion [135]. Selenium may influence embryo implantation in multiple ways [136,137]. First, oxidative stress can damage the embryo and uterine endometrium, leading to implantation failure [138]. In contrast, selenium’s antioxidant function reduces oxidative stress in the uterine microenvironment, preventing harm to the embryo’s survival and development [139,140]. Studies indicate, homozygous selenoprotein I knockout mouse embryos terminate development before E6.5 and fail to successfully implant in the uterus [141]. Second, selenium-regulated immune function helps maintain normal immune recognition function in the uterus [27]. It can recognize the embryo as its own cells, thereby preventing the embryo from encountering an immune rejection response during implantation [142,143]. Additionally, selenium may influence the expression of adhesion molecules and cytokines in the endometrium. For example, it can regulate the expression of integrins, which are key adhesion molecules involved in the process of embryo attachment to the endometrium, a critical interaction during implantation [144,145]. Leukemia inhibitory factor (LIF) is a cytokine crucial for embryo implantation. LIF participates in the process of endometrial decidualization during pregnancy and embryo implantation into the endometrium [146]. Selenium can increase LIF production, thereby creating conditions favorable for embryo attachment and invasion [147] (Figure 3).

### 4.2. Pregnancy Maintenance

During pregnancy, the growth and development of the fetus require a stable intrauterine environment. Selenium plays a role in this process in multiple ways. First, it helps maintain normal placental function and nutrient transfer, selenium deficiency in pregnant mice leads to restricted fetal growth [148]. Selenium-containing enzymes in the placenta reduce the production of ROS, protecting the placenta from oxidative stress damage and ensuring its normal physiological function [149]. Additionally, selenium helps regulate hormonal balance during pregnancy. Hormones such as progesterone maintain pregnancy by inhibiting uterine contractions and promoting endometrial growth [150]. Selenium can participate in regulating the synthesis, metabolism, and action of these progesterone hormones, thereby maintaining pregnancy. As pregnancy progresses, blood selenium levels gradually decrease [151]. An earlier study showed that mean serum selenium values were significantly higher in early pregnancy (109 μg/L) than in late pregnancy (85.3 μg/L) in a study of pregnant Spanish women [152]. Recent studies have also shown that serum selenium concentrations are significantly lower in pregnant women than in non-pregnant women, and that selenium levels are lower in multiparous than in primiparous women [153]. Therefore, in some animal experiments, selenium deficiency led to abnormal progesterone levels in animals, resulting in increased uterine contractions during pregnancy and significantly increasing the risk of miscarriage [81,154]. Through transcriptomic sequencing analysis, the study found that maternal selenium deficiency inhibits progesterone biosynthesis by suppressing the expression of Hsd3b1 gene [155]. Selenium can also regulate the expression of genes involved in placental development and function [148]. For example, vascular endothelial growth factor (VEGF) promotes blood vessel formation in the placenta and ensures the transport of nutrients and oxygen from the mother to the fetus [156,157]. Selenium can regulate VEGF synthesis, ensuring adequate nutrient and oxygen supply to the placenta [158]. Other studies have also shown that selenium can mitigate the toxicity of chromium poisoning in pregnant rats and reduce pathological damage to the placenta [159]. Selenium deficiency in pregnant mice leads to dysregulation of placental nutrient transporters and fetal growth restriction [148].

### 4.3. Parturition

The effects of selenium on the uterus of female animals are also evident during the birthing process. Selenium may influence the contractility of uterine smooth muscle, thereby affecting the expulsion of the fetus during childbirth. Research indicates that selenium deficiency can lead to abnormal contractions of uterine smooth muscle in animals [160]. Smooth muscle contraction is regulated by the RhoA/Rho-associated protein kinase (ROCK) signaling pathway [161]. ROCK protein kinase can phosphorylate and activate myosin light chain kinase (MLCP), promoting myofibril contraction and thereby regulating smooth muscle contraction and vascular tone [162]. Selenium deficiency alters the activity of this signaling pathway. Myosin light chain (MLC) is essential for smooth muscle contraction. In selenium-deficient animals, MLCP activity decreases, resulting in reduced MLC phosphorylation, which leads to decreased smooth muscle contraction force and frequency [163]. Adequate selenium levels in the body maintain the normal function of this signaling pathway and ensure normal uterine contractions during labor. On the other hand, selenium influences the synthesis and metabolism of prostaglandins (PGs) during labor [164,165]. PGs play a key role in labor initiation and uterine contraction regulation, causing uterine contractions and involution. Selenium promotes normal labor in rabbits by regulating the expression of enzymes involved in PGs synthesis, such as cyclooxygenase-2 (COX-2) [166] (Figure 3), which affects the levels and activity of PGs within the uterus [167]. Selenium supplementation plays a positive role in maintaining pregnancy and promoting labor. However, selenium has only a limited effect in promoting labor and should only be used as a supplemental additive. During gestation in female mammals, selenium can be supplemented at appropriate doses to support the body’s synthesis of progesterone.

## 5. Selenium and Uterine Diseases

### 5.1. Endometritis

**Figure 4:**
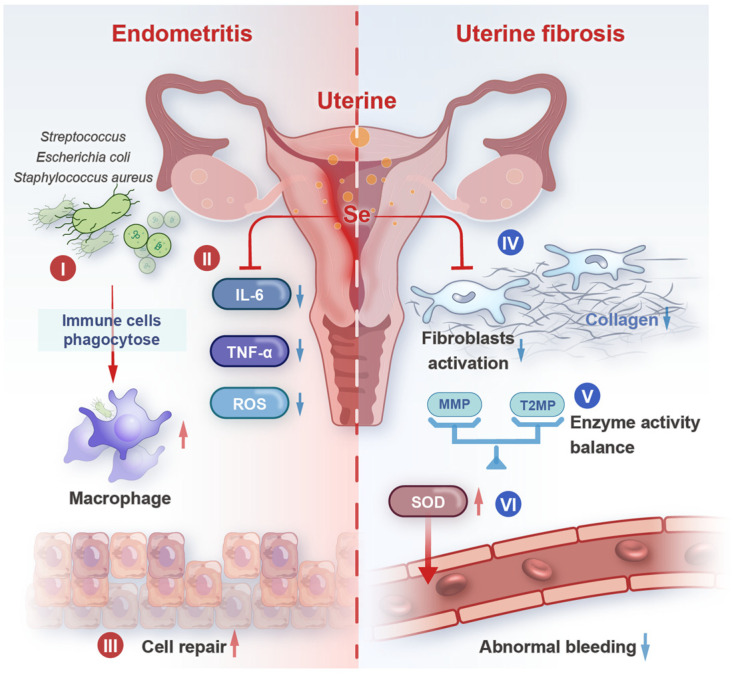
Selenium has healing and preventive effects on uterine diseases.

Endometritis is a common uterine disease in female animals, especially dairy cows and sows, and may be caused by bacterial infections such as *Escherichia coli*, *Staphylococcus aureus*, and *Streptococcus* spp. Selenium plays a significant role in both the prevention and treatment of endometritis [168]. As mentioned earlier, selenium’s antioxidant and anti-inflammatory properties are effective in mitigating the damage caused by oxidative stress and inflammation in the endometrium during bacterial infections [41,169]. Research indicates that supplementation with selenomethionine (MSC) and methylselenate (MSA) can reduce the production of proinflammatory cytokines, providing significant protective effects against Staphylococcus aureus-induced endometritis in rats [170] (Figure 4). Moreover, selenium can enhance immunity, helping animals resist bacterial infections and thereby reducing the incidence and severity of endometritis [87]. Pre- and postpartum supplementation of Holstein cows with 0.3 mg/kg sodium selenite or selenium-enriched yeast, as a result, can reduce the incidence of subacute endometritis in dairy cows [171]. For example, some studies have shown that sows supplemented with selenium-rich yeast have significantly better antioxidant status than sows without selenium-supplementation [172]. Oral administration of selenium yeast to goats, in addition to feeding them a diet containing 0.6 mg/kg selenium, effectively reduces endometrial inflammatory responses induced by Escherichia coli under conditions of elevated cortisol levels [169,173]. At the same time, the same conclusion was reached in in vitro experiments supplementing selenium to bovine endometrial epithelial cells [174]. Antimicrobial peptides, such as defensins, have direct antibacterial activity against invading pathogens [175]. An increase in these peptides helps selenium better help the endometrium resist bacterial infection, reducing the risk of endometritis.

### 5.2. Uterine Fibrosis

Endometrial fibrosis is a condition of the endometrium characterized by excessive deposition of extracellular matrix proteins (such as collagen) in the uterus, which can impair uterine function and fertility [176]. Fibroblasts are cells responsible for producing extracellular matrix proteins, and oxidative stress can activate fibroblasts [177]. Selenium can reduce oxidative stress and inhibit fibroblast activation [178,179]. Thus, selenium reduces the expression of genes associated with intracellular collagen synthesis, the excessive production of collagen and other extracellular matrix components [180,181], preventing endometrial fibrosis. Additionally, selenium’s anti-inflammatory effects can alleviate uterine inflammation, thereby reducing fibroblast activation and extracellular matrix deposition [182]. Within cells, tissue metalloproteinase inhibitors (TIMPs) regulate the physiological degradation and remodeling of the extracellular matrix by forming a dynamic equilibrium with matrix metalloproteinases (MMPs) [131,183]. MMPs are responsible for the degradation of extracellular matrix proteins, while TIMPs inhibit the activity of MMPs in uterine tissue [184,185]. Selenium can regulate the activity of MMPs and TIMPs [131], maintaining the balance between MMPs and TIMPs in uterine tissue, thereby regulating the production and metabolism of the extracellular matrix, preventing its excessive deposition, and preventing endometrial fibrosis [186,187] (Figure 4).

### 5.3. Retained Placenta

Retained placenta refers to the failure of a female animal to expel the placental membranes within a specific timeframe after calving. It is a common reproductive disorder in livestock such as dairy cows and sows [188]. This condition not only leads to endometritis, delayed conception, and reduced lactation performance but also causes significant economic losses. Scientific research has conclusively demonstrated that selenium deficiency is a key nutritional factor contributing to retained placenta [189], with its mechanism primarily linked to the antioxidant functions of selenium and alpha-tocopherol [190]. Studies indicate that concurrent supplementation of selenium and vitamin E in Holstein dairy cows effectively reduces the incidence of retained placenta [191]. In selenium-deficient regions of Norway, selenium supplementation can reduce the incidence of retained placenta in dairy herds [192]. Supplementing organic selenium in the diets of pregnant livestock allows it to be transferred to the fetus via the placenta [193], increasing selenium levels in colostrum and effectively reducing the incidence of retained placenta. Whether administered via intramuscular injection or oral supplementation, selenium can reduce the incidence of retained placenta in dairy cows [194]. Therefore, supplementing selenium and vitamin E before the birth of offspring is essential to prevent significant economic losses.

### 5.4. Other Uterine Diseases

In addition to endometritis and uterine fibrosis, selenium may be associated with other uterine disorders. For example, selenium’s function in regulating blood vessels and aspects of its antioxidant activity can play a role in certain uterine bleeding disorders [195,196]. Oxidative stress can damage uterine blood vessels, leading to abnormal uterine bleeding. However, GPXs can prevent and treat abnormal uterine bleeding by protecting the vessel wall from damage and maintaining vascular integrity and normal function [197]. In addition, supplementation with selenium and vitamin E maintains redox balance and prevents iron death and inflammation in lymphocytes within the intestinal epithelium [198]. Although research in this area is still relatively limited, it suggests that selenium has great potential for maintaining overall uterine health and preventing various uterine diseases. In addition, selenium regulates intracellular calcium levels and the activity of smooth muscle contraction-related proteins [199,200], restoring normal uterine smooth muscle function and preventing the symptoms of weak contractions during labor [201] (Figure 4). These uterine diseases can all lead to reduced reproductive performance in animals. Therefore, in practical production settings, selenium supplementation can be used to lower the risk of disease. However, current research on selenium-containing drugs remains limited, with most studies focusing on anti-cancer applications and alleviate oxidative stress [202,203]. It is hoped that future research will increasingly concentrate on selenium therapy for diseases affecting female mammals.

## 6. Conclusions

Selenium is an essential trace element for mammals, supporting uterine function through multiple physiological mechanisms. These mechanisms include antioxidant, anti-inflammatory, anti-apoptotic, and immunomodulatory actions. Specifically, selenium promotes normal uterine development and structural formation, ensuring successful embryo implantation and pregnancy maintenance, while regulating uterine smooth muscle contractions during labor. Selenium also prevents and treats common uterine disorders such as endometritis and uterine fibrosis.

Meanwhile, we found that there are significant differences in the bioavailability and safety of different forms of selenium sources. Organic selenium shows better application prospects in improving uterine health and reproductive performance in animals due to its higher absorption efficiency and lower risk of toxicity. Current research on the effects of selenium on the mammalian uterus has been limited to animals that bring economic effects such as cattle, sheep, and pigs, and little research has been done on companion animals such as dogs and cats. We hope that more studies focusing on different species will fill this gap in the future. Future research should further clarify the differences in selenium requirements of different animal species at different physiological stages, explore the synergistic mechanism between selenium and other nutrients, and optimize selenium supplementation strategies. This aims to achieve the goal of precise regulation of animal uterine health and enhancement of breeding efficiency. The development of selenium-containing supplements for female mammals should be pursued to improve production and treat diseases. In-depth exploration of the molecular mechanisms of selenium in the field of uterine health will provide important theoretical support for the development of new animal reproductive health technologies and green feed additives. It can promote the development of animal husbandry in the direction of high efficiency, health and sustainability.

## Figures and Tables

**Table 1:** Effects of organic and inorganic selenium sources on the uterus of different animals.

Species	Organic Selenium	Inorganic Selenium
Hens [26]	Bacterial organic selenium supplementation promotes eggshell mineralization and increases blood selenium concentrations.	Supplementation with inorganic selenium also increased antioxidant enzyme levels, but GPX3/GPX4 levels were much lower than supplementation with organic selenium.
Cows [27]	Conceptus length was increased in heifers supplemented with MIX (both ISe and Ose) (25.96 ± 3.95 cm) compared with ISe (17.45 ± 3.08 cm).	Glucose in amniotic fluid of ISe-supplemented ewes was less than in the MIX group.
Rats [28]	Selenium concentration in placenta samples from the selenium nanoparticle group was 2-fold higher than that in placenta samples from the sodium selenate-treated group.	Body weights of pregnant rats in the SeNPs group were lower than those in the NaSe group at day 21.
Pigs [29]	Increase in litter weight of piglets in HMSeBA-supplemented dams during pregnancy compared to the Na_2_SeO_3_ group.	Reduced elevation of maternal antioxidant enzyme activities in the Na_2_SeO_3_ supplemented group compared to HMSeBA supplemented during pregnancy.

## Data Availability

All the available data are presented in the article.
